# The bottleneck for maternal transmission of mtDNA is linked to purifying selection by autophagy

**DOI:** 10.1126/sciadv.aea4660

**Published:** 2025-11-12

**Authors:** Laura S. Kremer, Zoe Golder, Tom Barton-Owen, Polyxeni Papadea, Camilla Koolmeister, Patrick F. Chinnery, Nils-Göran Larsson

**Affiliations:** ^1^ Division of Molecular Metabolism, Department of Medical Biochemistry and Biophysics, Karolinska Institutet, Stockholm, Sweden.; ^2^ Department of Cellular Biochemistry, University Medical Center Göttingen, 37073 Göttingen, Germany.; ^3^ MRC Mitochondrial Biology Unit, School of Clinical Medicine, University of Cambridge, Cambridge, UK.; ^4^ Department of Clinical Neuroscience, School of Clinical Medicine, University of Cambridge, Cambridge, UK.

## Abstract

Mammalian mitochondrial DNA (mtDNA) inheritance differs fundamentally from nuclear inheritance owing to exclusive maternal transmission, high mutation rate, and lack of recombination. Two key mechanisms shape this inheritance: the bottleneck, which drives stochastic transmission of maternal mtDNA variants, and purifying selection, which actively removes mutant mtDNA. Whether these mechanisms interact has been unresolved. To address this question, we generated a series of mouse models with random mtDNA mutations alongside alleles altering mtDNA copy number or decreasing autophagy. We demonstrate that tightening the mtDNA bottleneck increases heteroplasmic variance between individuals, causing lower mutational burden and nonsynonymous-to-synonymous ratios. In contrast, reduced autophagy weakens purifying selection, leading to decreased interoffspring heteroplasmic variance and increased mutational burden with higher nonsynonymous-to-synonymous ratios. These findings provide experimental evidence that the mtDNA bottleneck size modulates the efficacy of purifying selection. Our findings yield fundamental insights into the processes governing mammalian mtDNA transmission with direct implications for the origin and propagation of mtDNA mutations causing human disease.

## Teaser

Revealing mechanisms of mtDNA transmission is critical for combating human disease.

## INTRODUCTION

Mammalian mitochondrial DNA (mtDNA) is exclusively inherited from the mother without undergoing recombination (*1*–*3*) and has a very high mutation rate in comparison with nuclear genes (*4*). Low-frequency mtDNA mutations have been detected in most healthy humans including female germ cells (*5*–*7*), and at least 1:250 healthy humans carry a pathogenic mtDNA mutation with an allele frequency of >10% (*8*–*10*). The allele frequency of mtDNA is typically referred to as the heteroplasmic fraction (HF) because the high copy number and maternal inheritance distinguish mtDNA variants from nuclear DNA variants. If transmitted to the next generation, mtDNA mutations can lead to mitochondrial disorders and contribute to age-associated diseases, such as heart failure, cancer, neurodegeneration, and diabetes, and influence the aging process itself (*11*–*16*). Despite the importance for human pathology, the principles for maternal transmission of mammalian mtDNA are incompletely understood. The strict asexual mode of mtDNA transmission is predicted to lead to irreversible mutation accumulation, the so-called Muller ratchet phenomenon (*17*, *18*). At least two main mechanisms in the maternal germline are thought to counteract a mutational meltdown: a reduction in mtDNA content within germ cells (the mtDNA bottleneck), which leads to rapid segregation of heteroplasmic variants and the stochastic transmission of only a subset of all mtDNA variants present in the mother (*19*), and purifying selection that actively suppresses the transmission of deleterious mtDNA mutations (*20*, *21*). Despite their importance, it has remained unclear whether both mechanisms act independently or whether they are functionally linked (*22*). To address this question, we generated a large set of mouse models with random mtDNA mutations and introduced nuclear mutations that decrease (*23*) or increase mtDNA copy number (*24*), or potentially modulate selection by impairing autophagy (*25*). In adult mice, the effects of pathogenic mtDNA mutations are ameliorated by increased mtDNA copy number in some differentiated tissues (*26*, *27*) whereas other tissues remain unaffected (*28*). Autophagy is reported to play an important role in mediating purifying selection of mtDNA in worms and fruit flies (*29*–*31*) and has been shown to affect transmission of the m.5024C>T mutation in the tRNA^Ala^ gene in mice (*32*). However, the link between mtDNA copy number, autophagy, and germline transmission of mtDNA mutations in mammals has previously not been systematically studied. In the present study, we show that a reduction of the mtDNA bottleneck size increases the variance in heteroplasmy levels between individuals and is linked to a concurrent lowering of the mutational burden and the nonsynonymous-to-synonymous (N/S) variant ratio. Furthermore, we show that reduced autophagy weakens purifying selection, leading to a decreased interoffspring heteroplasmic variance, increased mutational burden, and elevated N/S variant ratios. Thus, the size of the mtDNA bottleneck modulates the strength of purifying selection, providing fundamental insights into the processes governing mammalian mtDNA transmission with direct implications for the origin and propagation mtDNA mutations causing human disease.

## RESULTS

### Inheritance of random mtDNA mutations in the mouse recapitulates human mtDNA transmission

**Fig. 1.**
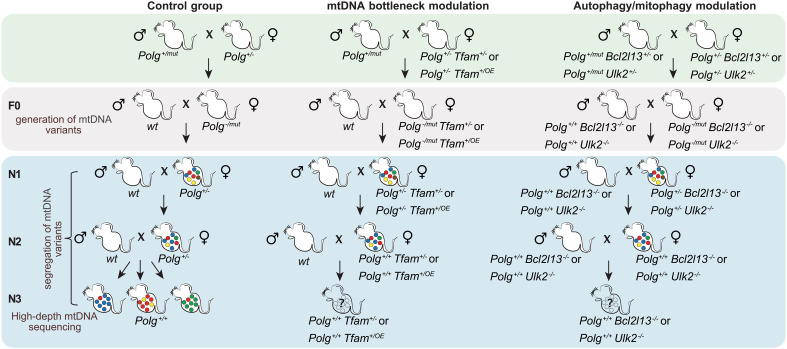
Schematic representation of the breeding schemes. Mating strategy to generate mice transmitting random mtDNA variants in combination with modulation of the mitochondrial transcription factor A (*Tfam^+/−^* or *Tfam^+/OE^*) for mtDNA bottleneck modulation or in combination with knockouts of B cell CLL/lymphoma 2–like 13 (*Bcl2l13^−/−^*) or unc-51–like autophagy activating kinase 2 (*Ulk2^−/−^*) alleles for modulating the autophagic turnover of mitochondria.

We generated DNA polymerase gamma (*Polg*)–mutator mice, which randomly generate mtDNA mutations (*20*, *33*), by crossing heterozygous knockout (*Polg*^+/−^) females to heterozygous mutator (*Polg*^+/mut^) males. The resulting hemizygous females (*Polg*^−/mut^) only generate mtDNA variants de novo during embryonic and postnatal life and do not contain increased levels of preexisting maternally transmitted variants. The use of hemizygous *Polg*-mutators enabled easy removal of the *Polg*-mutator allele by breeding in the subsequent generation, thereby restricting increased mtDNA mutagenesis exclusively to a single generation (F0). The generated mtDNA mutations arise de novo on a single mtDNA molecule, and segregation over successive generations is necessary to reach an HF at which purifying selection can be assessed. As previous studies have shown that selection readily becomes detectable by the third generation (*34*), we examined transmission from the N2 to the N3 generation before and after genetically modifying nuclear genes that modulate mtDNA levels or autophagy (Fig. 1).

We studied the effect of modulating mtDNA copy number by reducing or increasing the expression of the mitochondrial transcription factor A (*Tfam*^+/−^, *Tfam*^+/OE^) (Fig. 1, middle). We also reduced autophagy by knocking out the unc-51–like autophagy activating kinase 2 (*Ulk2*^−/−^) (*25*), an inducer of bulk autophagy, or B cell CLL/lymphoma 2 like 13 (*Bcl2l13*^−/−^) (*32*, *35*), involved in targeted removal of mitochondria (Fig. 1, right), both of which are expressed during female germ cell development (*32*). We subsequently generated very high–depth mtDNA sequence data (*34*) from N3 mice across all groups to assess the impact on the mtDNA mutational burden.

**Fig. 2.**
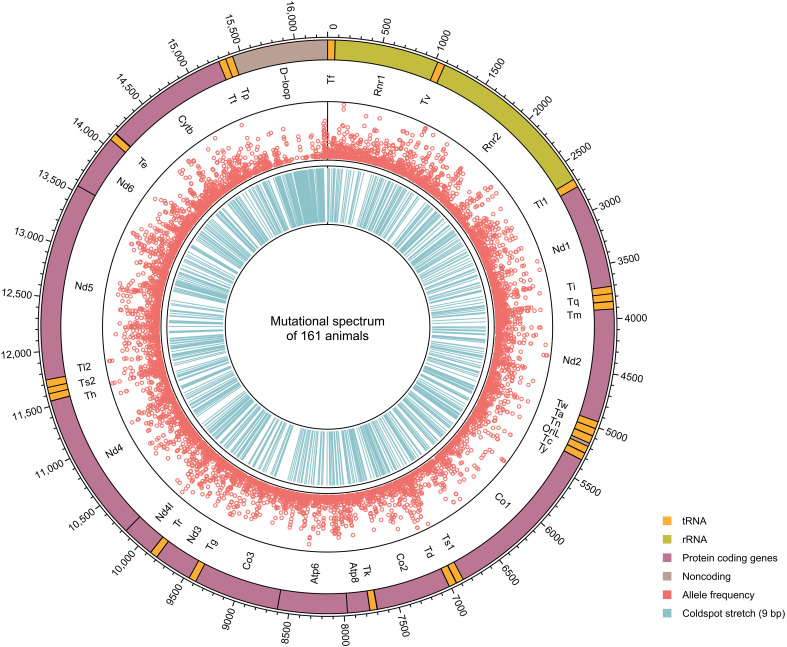
Circos plot of mitochondrial variants identified in 161 mice. Circles from the outside to the inside show (i) regions and names of the different mtDNA genes (orange, tRNA; chartreuse, rRNA; pink, protein coding genes; and brown, noncoding); (ii) allele frequencies [heteroplasmic fraction (HF)] of the detected mtDNA variants (the radial axis corresponds to the HF); and (iii) coldspot stretches without any variants for at least nine adjacent positions.

**Fig. 3.**
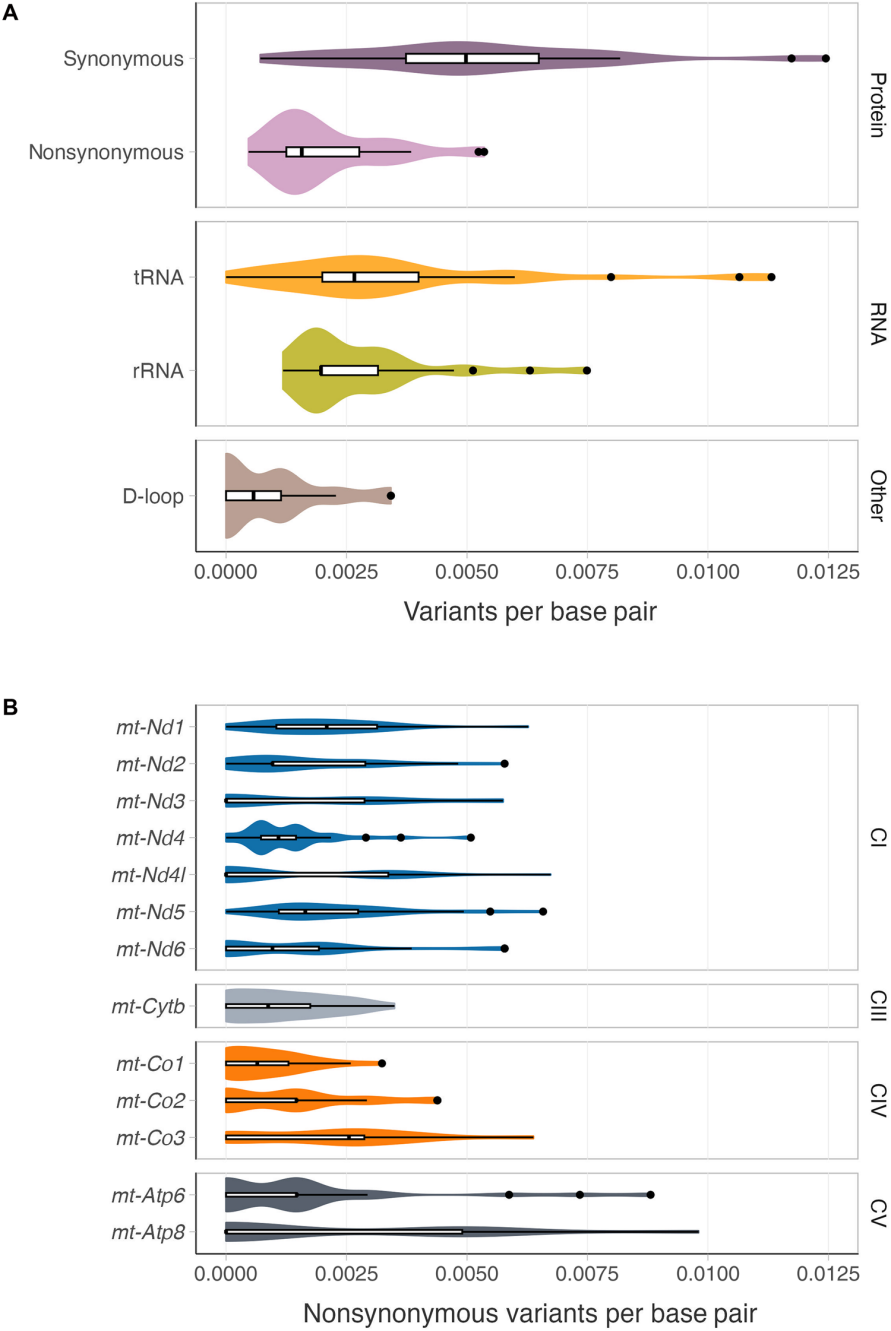
Evidence of purifying selection acting on inherited mtDNA variants. (**A**) Number of variants per base pair for the different mtDNA genes (dark pink, synonymous variants in protein coding genes; light pink, nonsynonymous variants in protein coding genes; orange, variants in tRNA genes; chartreuse, variants in rRNA genes; and brown, variants in the noncoding region). (**B**) Number of variants per base pair for the different protein coding genes of the mtDNA [blue, mtDNA genes encoding oxidative phosphorylation (OXPHOS) system complex I subunits; light gray, mtDNA genes encoding OXPHOS complex III subunits; dark orange, mtDNA genes encoding OXPHOS complex IV subunits; and dark gray, mtDNA genes encoding OXPHOS complex V subunits].

In total, we sequenced mtDNA of 161 mice from five different nuclear backgrounds, namely, *Polg*^+/+^, *Polg*^+/+^*Tfam*^+/−^, *Polg*^+/+^*Tfam*^+/OE^, *Polg*^+/+^*Bcl2l13*^−/−^, or *Polg*^+/+^*Ulk2*^−/−^ (Table 1). Overall, we detected 3366 unique variants at 3176 different positions (Fig. 2). The variants were found in all functional coding classes of the mitochondrial genome, including tRNAs, rRNAs, and mRNAs, as well as in the noncoding region. This comprehensive representation enabled an unbiased assessment across the whole mtDNA genome. In the *Polg*^+/+^ control group, we observed less nonsynonymous variants compared to synonymous variants (Fig. 3A) with the strongest effect for the mitochondrially encoded cytochrome *c* oxidase I (*mt-Co1*) and the weakest effect for the mitochondrially encoded ATP synthase 8 (*mt-Atp8*) and 6 (*mt-Atp6*) (Fig. 3B). These observations are consistent with purifying selection acting during transmission and mirror the situation in humans (*36*), endorsing our experimental approach.

**Table 1.** Number of mice per genotype group.

Genotype of N3 mice	Number of N3 mice
*Polg^+/+^*	32
*Polg^+/+^ Tfam^+/−^*	32
*Polg^+/+^Tfam^+/OE^*	31
*Polg^+/+^Bcl2l13^−/−^*	34
*Polg^+/+^ Ulk2^−/−^*	32

### Copy number and autophagy shape mtDNA inheritance

**Fig. 4.**
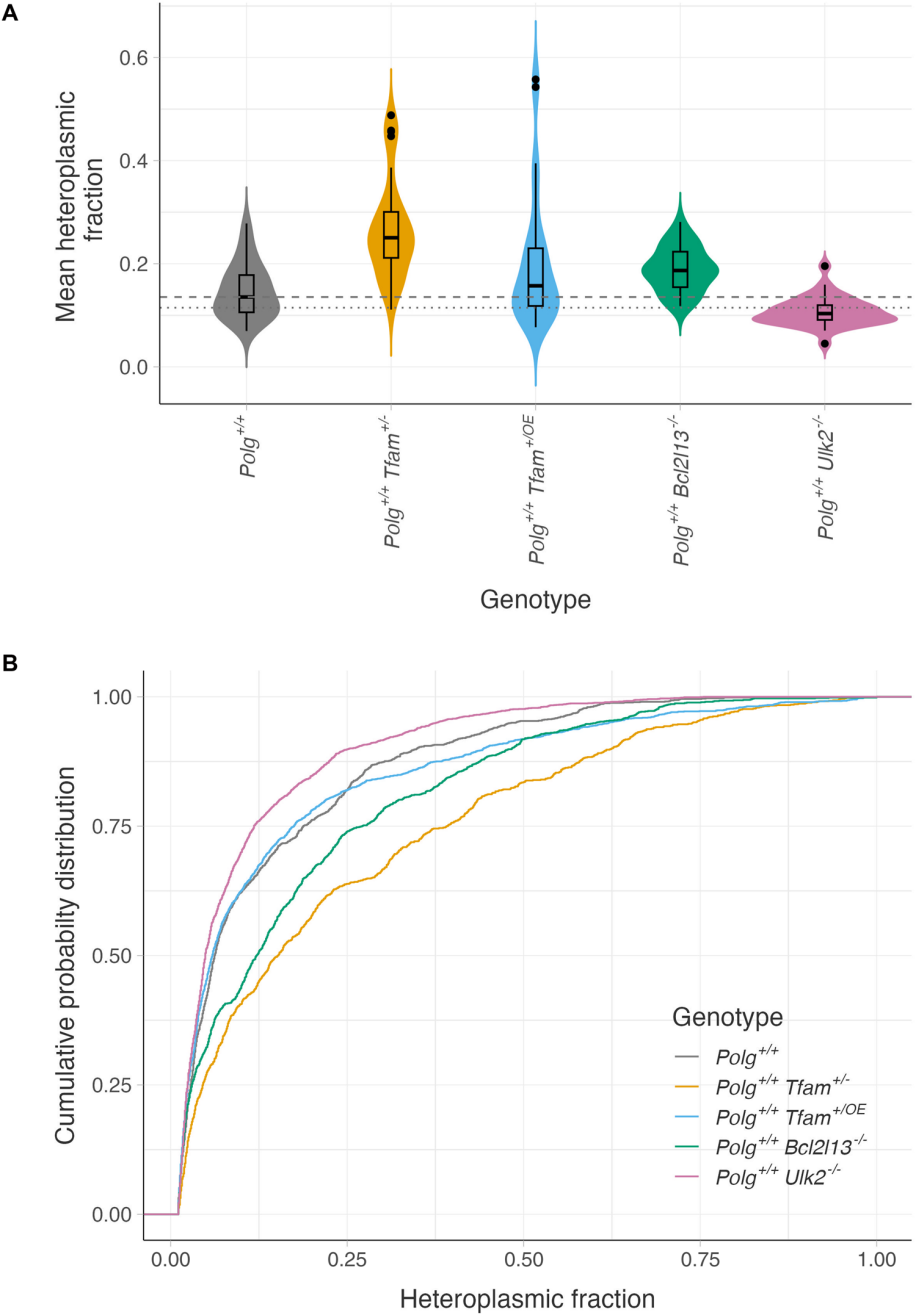
Distinct effects of bottleneck modulation and impairment of autophagy on the distribution of mtDNA heteroplasmy. (**A**) Mean HF was calculated for each animal by averaging the HF of all detected mtDNA variants in that individual. Dashed line, median value of the *Polg*^+/+^ control group; dotted line, modal value of the *Polg*^+/+^ control group; gray, *Polg*^+/+^ control group; orange, *Polg*^+/+^ *Tfam*^+/−^ group; blue, *Polg*^+/+^ *Tfam*^+/OE^ group; green, *Polg*^+/+^ *Bcl2l13*^−/−^ group; and pink, *Polg*^+/+^ *Ulk2*^−/−^ group. (**B**) Cumulative distributions of the HF are shown for each experimental group, illustrating differences in the overall distribution of mtDNA variant frequencies. Gray, *Polg*^+/+^ control group; orange, *Polg*^+/+^ *Tfam*^+/−^ group; blue, *Polg*^+/+^ *Tfam*^+/OE^ group; green, *Polg*^+/+^ *Bcl2l13*^−/−^ group; and pink, *Polg*^+/+^ *Ulk2*^−/−^ group.

To determine whether mtDNA levels and autophagy influence the inheritance of mtDNA variants, we estimated the mean HF across the detected variants within each animal and computed for each group the median, being the “central” value, and the mode, which is the most frequently observed value. We subsequently used Wilcoxon’s rank test to compare the median values of *Polg*^+/+^*Tfam*^+/−^, *Polg*^+/+^*Tfam*^+/OE^, *Polg*^+/+^*Bcl2l13*^−/−^, or *Polg*^+/+^*Ulk2*^−/−^ groups to *Polg*^+/+^ controls. To the best of our knowledge, no suitable statistical test exists for comparing modal values. This approach was similarly applied to all other parameters analyzed throughout the study. In comparison to the *Polg*^+/+^ control group (HF median = 0.14; HF mode = 0.11), we observed a strong HF increase in the *Polg*^+/+^*Tfam*^+/−^ group (HF median = 0.25, *P* = 0.0000000137; HF mode = 0.24) and a milder effect for the knockout of *Bcl2l13* (HF median = 0.19, *P* = 0.000345; HF mode = 0.19) (Fig. 4A). In contrast, *Polg*^+/+^*Ulk2*^−/−^ animals showed a decrease in the HF (HF median = 0.10, *P* = 0.000473; HF mode = 0.09) while we did not detect any difference in HF for the *Polg*^+/+^*Tfam*^+/OE^ group (HF median = 0.16, *P* = 0.168; HF mode = 0.13). The increasing effects on HF seen in the *Polg*^+/+^*Tfam*^+/−^ and *Polg*^+/+^*Bcl2l13*^−/−^ animals and the decreasing effect on HF observed in the *Polg*^+/+^*Ulk2*^−/−^ group were reflected in the HF distribution (Fig. 4B), demonstrating that reduction of TFAM expression and disruption of BCL2L13 and ULK2 expression modulate the HF.

**Fig. 5.**
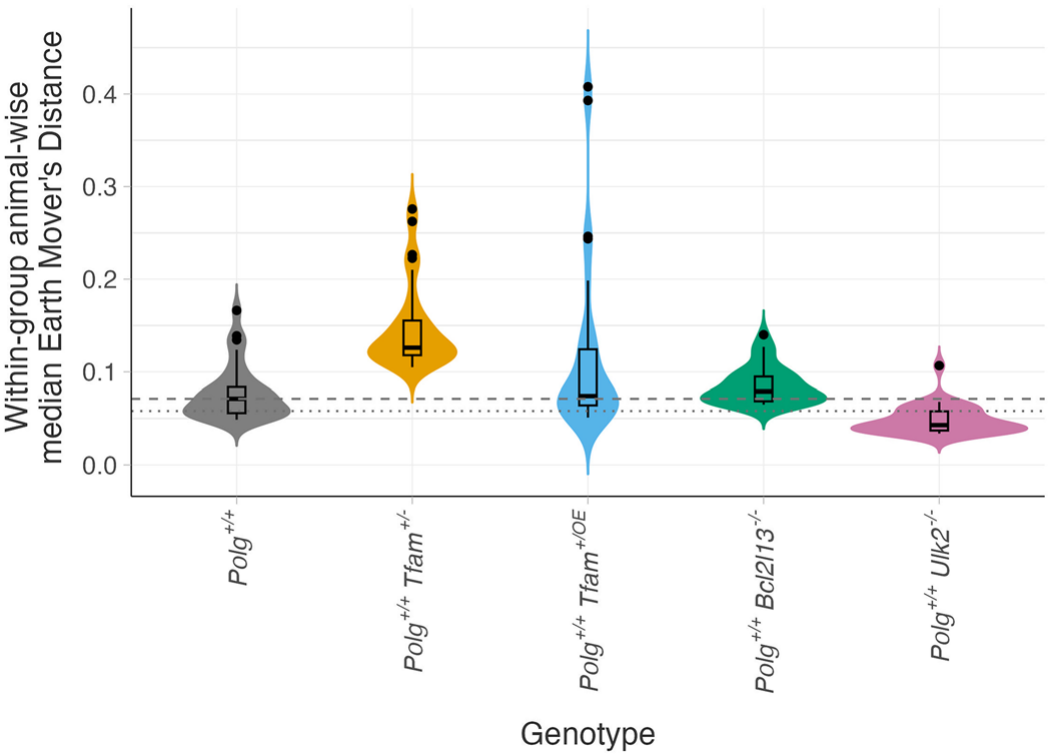
Mitochondrial bottleneck size and turnover shape interindividual heteroplasmy. Interindividual heteroplasmic differences within each experimental group were quantified using the Earth Mover’s Distance (EMD), a metric that captures the overall distributional shift of heteroplasmy between individual samples. Higher EMD values reflect greater variance in heteroplasmic profiles among mice within the same group. Dashed line, median value of the *Polg*^+/+^ control group; dotted line, modal value of the *Polg*^+/+^ control group; gray, *Polg*^+/+^ control group; orange, *Polg*^+/+^ *Tfam*^+/−^ group; blue, *Polg*^+/+^ *Tfam*^+/OE^ group; green, *Polg*^+/+^ *Bcl2l13*^−/−^ group; pink, *Polg*^+/+^ *Ulk2*^−/−^ group.

The elevated HF observed in the *Polg*^+/+^*Tfam*^+/−^ mice is consistent with a more stringent mtDNA bottleneck leading to a greater range of heteroplasmy values among offspring (*37*, *38*). Similarly, the reduced HF detected in the *Polg*^+/+^*Ulk2*^−/−^ mice points at a decreased variance in heteroplasmy values, which is in line with predictions that a general decrease in mtDNA turnover lowers heteroplasmic variance (*39*). To directly examine the variance in HF between each group, we quantified the differences in HF among animals within each group using the Earth Mover’s Distance (EMD). The EMD quantifies the similarity between two probability distributions by measuring the minimal “work” required to transform one distribution into the other, as exemplified by moving piles of earth. The *Polg*^+/+^ control group had a median EMD of 0.07 and an EMD mode of 0.06 (Fig. 5). In comparison, the EMD was strongly increased in the *Polg*^+/+^*Tfam*^+/−^ animals (EMD median = 0.13, *P* = 0.00000000688; EMD mode = 0.12), mildly increased in the *Polg*^+/+^*Bcl2l13*^−/−^ group (EMD median = 0.08, *P* = 0.0252; EMD mode = 0.07), decreased in the *Polg*^+/+^*Ulk2*^−/−^ group (EMD median = 0.04, *P* = 0.000000860; EMD mode = 0.04), and not changed in *Polg*^+/+^*Tfam*^+/OE^ animals (EMD median = 0.07, *P* = 0.0948; EMD mode = 0.07). Our findings thus establish that reduction in the effective size of the mtDNA genetic bottleneck increases the intersample heteroplasmic variance, while decreased general, nonselective turnover of the mitochondrial population caused by a compromised core autophagic machinery exerts the opposite effect where limited variant segregation leads to a reduction of the heteroplasmic variance. In contrast, depleting factors such as BCL2L13 that specifically target mitochondria to the autophagosome impairs the selective removal of pathogenic mtDNA variants and slightly increases the variance, likely because the selective action is only operational on mitochondria with mtDNA variants having an HF exceeding a certain threshold. The abolished BCL2l13 expression thus allows higher heteroplasmy levels to remain, which increases the range of heteroplasmy values that are tolerated, leading to increased heteroplasmy variance.

### Functional coupling of the bottleneck and purifying selection

**Fig. 6.**
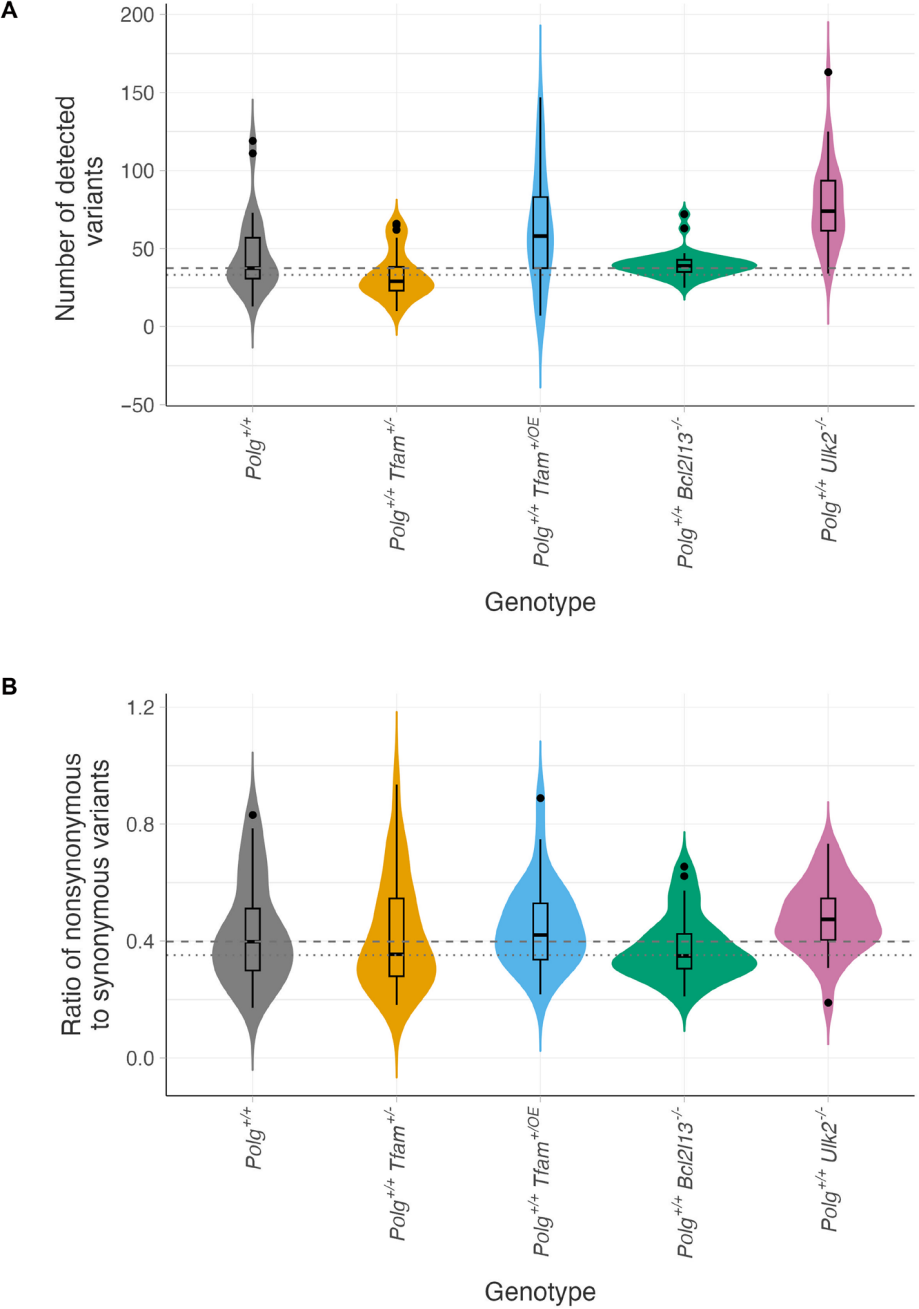
Altered bottleneck and mitochondrial turnover affect mtDNA mutation load and selection efficiency. (**A**) Mutational burden was quantified as the total number of mtDNA variants detected per animal. (**B**) For each animal, the mtDNA N/S ratio was calculated by dividing the number of nonsynonymous variants, normalized to the number of possible nonsynonymous variants in the mitochondrial genome, by the number of synonymous variants, normalized to the number of possible synonymous sites. For (A) and (B): dashed line, median value of the *Polg*^+/+^ control group; dotted line, modal value of the *Polg*^+/+^ control group; gray, *Polg*^+/+^ control group; orange, *Polg*^+/+^ *Tfam*^+/−^ group; blue, *Polg*^+/+^ *Tfam*^+/OE^ group; green, *Polg*^+/+^ *Bcl2l13*^−/−^ group; and pink, *Polg*^+/+^ *Ulk2*^−/−^ group.

We hypothesized that increased HF and increased variance will expose individual variants to selection mechanisms, whereas a lower HF and reduced variance should render individual variants less susceptible to selection. To test this assumption, we quantified the mutational burden, defined as the number of variants per mouse, within each group. In the *Polg*^+/+^ control animals, we observed a median burden of 37.5 and a modal value of 33.2 (Fig. 6A). Consistent with our assumption, the increased variance observed in the *Polg*^+/+^*Tfam*^+/−^ group was accompanied by a decrease in the mutational burden in these animals (median = 29.0, *P* = 0.0124; mode = 26.37). Furthermore, as predicted, the *Polg*^+/+^*Ulk2*^−/−^ group, characterized by low variance, exhibited an increased mutational burden (median = 74.0, *P* = 0.00000186; mode = 65.37). We also observed a higher mutational burden for *Polg*^+/+^*Tfam*^+/OE^ animals (median = 58.0, *P* = 0.0190; mode = 55.0) while the burden remained unchanged in the *Polg*^+/+^*Bcl2l13*^−/−^ group (median = 39.0, *P* = 0.878; mode = 39.15). Thus, reducing mtDNA levels enables stronger purifying selection and provides a direct link between the two processes. By contrast, selection is attenuated when general mitochondrial turnover is impaired.

To further corroborate our findings, we proceeded to calculate the N/S ratio of amino acid exchanges in protein coding genes. The *Polg*^+/+^ group had a median N/S ratio of 0.40 and an N/S ratio mode of 0.35 (Fig. 6B and fig. S1). While we did not observe a statistically significant deviation from the control median for any of the experimental groups (*Polg*^+/+^*Tfam*^+/−^ median = 0.36, *P* = 0.653; *Polg*^+/+^*Tfam*^+/OE^ median = 0.42, *P* = 0.346; *Polg*^+/+^*Bcl2l13*^−/−^ median = 0.35, *P* = 0.326; *Polg*^+/+^*Ulk2*^−/−^ median = 0.47, *P* = 0.0548), the *Polg*^+/+^*Tfam*^+/−^ animals showed a clearly lowered N/S ratio mode of 0.30. This is in line with a stronger purifying selection acting in the *Polg*^+/+^*Tfam*^+/−^ animals. In accordance with a weaker selection, the N/S ratio mode increased to 0.43 in *Polg*^+/+^*Ulk2*^−/−^ animals. The *Polg*^+/+^*Bcl2l13*^−/−^ group (mode 0.32) and the *Polg*^+/+^*Tfam*^+/OE^ group (mode = 0.39) showed only mild alterations. These data confirm a direct link between the mtDNA bottleneck and purifying selection, with general autophagic turnover of mitochondria playing a key role in this selective process. In contrast, impairing selective autophagic removal of damaged mitochondria and increasing the mtDNA bottleneck have only marginal effects.

## DISCUSSION

Faithful transmission of genetic material is essential for species survival. The evolutionary stability of mammalian mtDNA is threatened by a high mutation rate concurring with uniparental inheritance and lack of recombination, risking mutational meltdown through a Muller’s ratchet effect. Our findings demonstrate that both the mtDNA bottleneck and autophagic mitochondrial turnover interplay as key mechanisms to maintain genome integrity across generations. By experimentally modulating bottleneck size and mitochondrial turnover independently in mice, we report that tightening the mtDNA bottleneck through TFAM reduction increases the mean HF and amplifies intersample heteroplasmic variance. The elevated HF and variance expose individual variants to selection, thereby strengthening purifying selection. In contrast, impairment of general autophagic mitochondrial turnover limits variant segregation and reduces variance, which compromises selection and increases the mutational burden. These results establish a direct mechanistic link between the bottleneck phenomenon, mitochondrial turnover, and purifying selection in mammals. Important mechanistic understanding of mtDNA inheritance also comes from studies in *Drosophila melanogaster* (*30*, *40*). However, the germline of fruit flies differs from that of mammals because it is maintained by continuously dividing stem cells in the gonad that produce oocytes throughout adult life. Furthermore, the mtDNA bottleneck is much wider in *D. melanogaster* than in mammals, and this difference prevents clonal expansion of mtDNA mutations in just a few generations (*41*, *42*). Despite these fundamental differences, our results highlight that other important features of mtDNA transmission processes are conserved across metazoa. We observed different effects when modulating general, nonselective autophagic turnover of the entire mitochondrial population versus selective autophagic turnover of mitochondria. Inhibiting general turnover by depleting ULK2, a factor critical for autophagosome formation, slows mitochondrial turnover and reduces clonal expansion of all mtDNA variants and is manifested as a lowering of the HF and impaired purifying selection, leading to an increase of the overall mutational burden. This effect will limit random segregation and thereby reduces the number of variants reaching high levels that are acted upon by selection, which, in turn, leads to an increase of the number of variants detected in N3 offspring. In contrast, knocking out *Bcl2l13*, which selectively tethers damaged mitochondria to the autophagosome, is expected to only act on pathogenic mtDNA variants at an HF high enough to trigger selection, with their inefficient removal resulting in an increased HF. As most variants remain unaffected, the overall effect on the total allele burden is small. The effect might become more pronounced in subsequent generations, where a higher HF could elicit stronger purifying selection. In addition, it is now evident that multiple autophagy pathways exist, and functional redundancy among them likely explains why knockout of a single autophagy component has less effects than decreasing overall autophagy.

To summarize, our findings have broad implications for understanding mitochondrial genome evolution, the inheritance of pathogenic mtDNA variants, and the etiology of pathologies associated with human mtDNA mutations. Defining these mechanisms is essential for the development of therapeutic strategies targeting mitochondrial pathologies in humans. Recent examples of pronuclear transfer, a reproductive therapy for women carrying pathogenic mtDNA variants, have reported that carryover of mutated mtDNA to children born after mitochondrial donation is a concern (*43*, *44*). Pharmacological modulation of autophagy or transient overexpression of autophagy factors may provide future avenues to limit the spread of mtDNA mutations after mitochondrial donation. Conversely, reducing the mtDNA content in the donor oocyte could accelerate selection against the transmitted mtDNA mutations. If correct, this would also open the opportunity for pharmacological prevention of mtDNA disease.

## MATERIALS AND METHODS

### Mouse models

The *Polg^+/mut^*, the *Polg^+/−^*, *Tfam^+/−^*, *Tfam^+/OE^*, *Bcl2l13^+/−^*, and *Ulk2^+/−^* mice were generated as previously described (*23*, *24*, *26*, *32*, *33*, *45*). All animals included in this study were on a C57BL/6NCrl nuclear background. The experiments were performed in accordance with the guidelines of the Federation of European Laboratory Animal Science Associations and approved by the Stockholm ethical committee (Stockholms djurförsöksetiska nämnd) under the ethical permit 2001–2018.

### High-depth mtDNA sequencing

Total DNA from tail biopsies from 8-week-old animals was isolated using the DNeasy 96 Blood & Tissue Kit (Qiagen 69581). Each mtDNA sample was amplified with two overlapping long-range polymerase chain reaction amplicons (*46*), which were then purified, quantified, and pooled in equal amounts. Libraries were prepared and indexed using NEBNext Ultra library prep reagents (NEB E7805, E7600, and E7780) and sequenced using a 2 × 250-cycle MiSeq Reagent kit v3.0 (Illumina).

### MtDNA variant analysis

The Fastq files generated were used for mitochondrial variant calling performed with the standard MToolBox pipeline (*47*). Annotation and pathogenicity prediction of mtDNA variants were performed using SnpEff (version 5.2c) (*48*). Downstream analyses were performed in R (version 4.4.2) (https://R-project.org) using the tidyverse (version 2.0.0) (*49*), stringr (version 1.5.1) (https://CRAN.R-project.org/package=stringr), and data.table (version 1.17.0) [Barrett T (2025) https://CRAN.R-project.org/package=data.table] packages. We restricted our analysis to variants with HF > 1% that were observed in fewer than 26 animals, thereby excluding any variants likely inherited from a subline rather than being generated de novo. Circular visualization of mtDNA variants was performed using the circlize (version 0.4.16) package (*50*). EMD was calculated using the transport (version 0.15-4) package (https://cran.r-project.org/package=transport). Statistical analysis was conducted using ggpubr (version 0.6.0) (https://CRAN.R-project.org/package=ggpubr) and figures were generated using ggplot2 (version 3.5.1) (*51*) and cowplot (version 1.1.3) (https://CRAN.R-project.org/package=cowplot). Data tables were exported to Excel format using the xlsx (version 0.6.5) package (https://cran.r-project.org/package=xlsx).

### Statistical analysis

The sample size is indicated in Table 1 and included at least 30 mice where statistical evaluation was performed. Normality of the data was assessed in R using the Shapiro-Wilk test, which indicated that the data were not normally distributed. Consequently, Wilcoxon’s rank tests were applied instead of parametric *t* tests using the ggpubr package ggpubr (version 0.6.0). It should be noted that Wilcoxon’s rank tests do not account for the nonindependence of pups from the same litter or dam, which can artificially inflate the effective sample size (*n*). Images were created with Adobe Illustrator 2020 and Affinity Designer.
