# Supplementary material for: The bottleneck for maternal transmission of mtDNA is linked to purifying selection by autophagy
Source: Sci Adv. 2025 Nov 12;11(46):eaea4660. doi: 10.1126/sciadv.aea4660 (PMC12609065; doi:10.1126/sciadv.aea4660)
Supplement: Supplementary file 1 — Fig. S1 Legend for table S1 [file sciadv.aea4660_sm.pdf]

Science Advances
AAAS

Supplementary Materials for

The bottleneck for maternal transmission of mtDNA is linked to purifying selection by autophagy

Laura S. Kremer *et al.*

Corresponding author: Laura S. Kremer, laura.kremer@med.uni-goettingen.de;
Nils-Göran Larsson, nils-goran.larsson@ki.se

Sci. Adv. **11**, eaea4660 (2025)
DOI: 10.1126/sciadv.aea4660

The PDF file includes:

Fig. S1
Legend for table S1

Other Supplementary Material for this manuscript includes the following:

Table S1

Supplementary Materials

Supplementary Figure 1

Supplementary Table 1.

S1

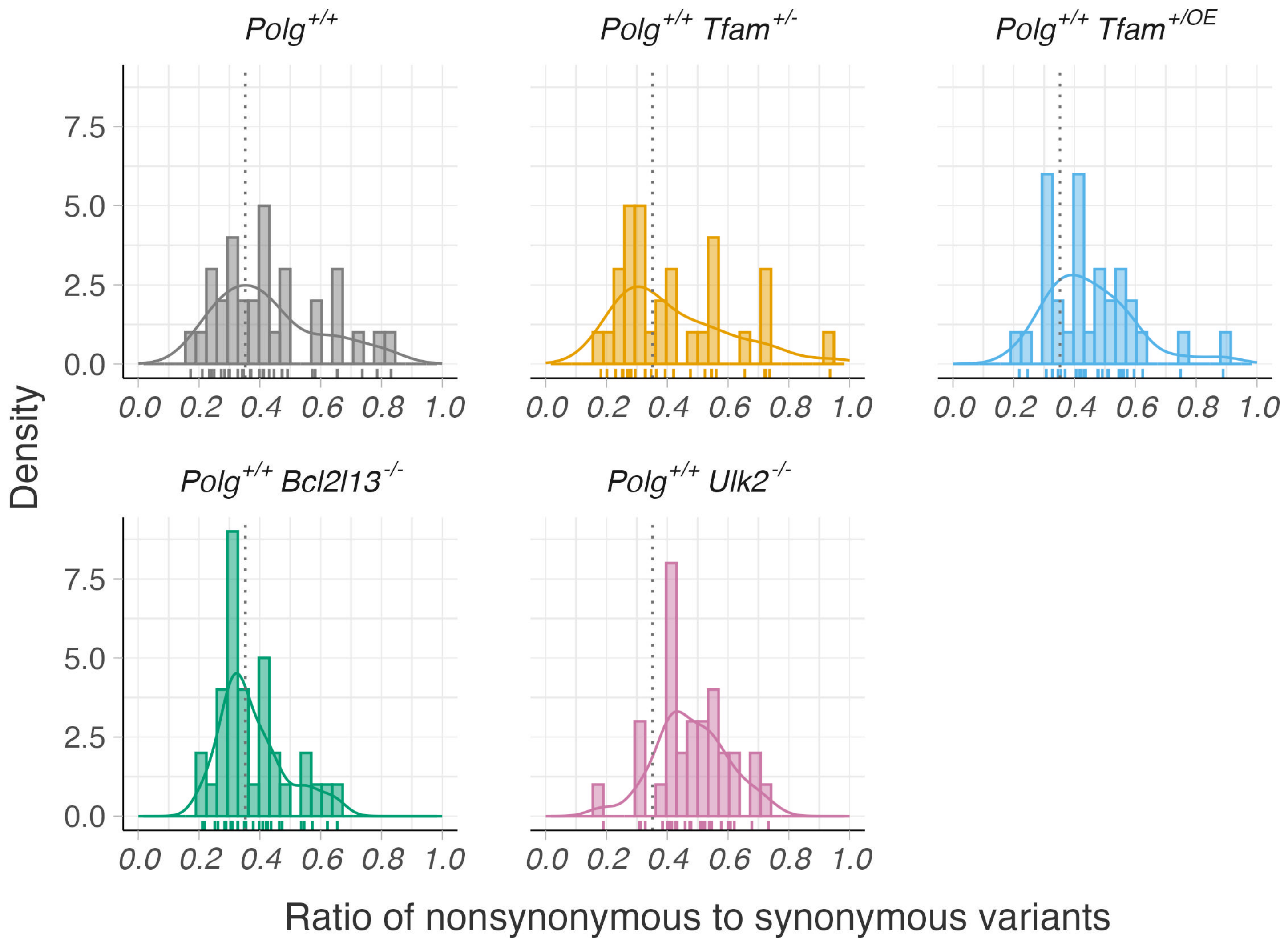

Supplementary Figure 1: Genotype-specific differences in the distribution of nonsynonymous-to-synonymous ratios across individual mice. Histograms of nonsynonymous-to-synonymous ratio in each mouse with fitted kernel density curves.

Supplementary Table 1. High-depth mtDNA sequencing results. Variants identified by high-depth mtDNA sequencing in mice of different genotypes.
